# Organic and Conventional Yerba Mate (*Ilex paraguariensis* A. St. Hil) Improves Metabolic Redox Status of Liver and Serum in Wistar Rats

**DOI:** 10.3390/antiox2030100

**Published:** 2013-07-24

**Authors:** Cátia S. Branco, Gustavo Scola, Adriana D. Rodrigues, Verónica Cesio, Horacio Heinzen, Alessandra Godoy, Cláudia Funchal, Adriana S. Coitinho, Mirian Salvador

**Affiliations:** 1 Institute of Biotechnology, University of Caxias do Sul, Caxias do Sul, RS 95070-560, Brazil; E-Mails: csbranc1@ucs.br (C.S.B.); gustavo.scola@gmail.com (G.S.); adry.dr@gmail.com (A.D.R.); 2 Department of Natural Products and Pharmacognosy, Faculty of Chemistry, University of the Republic, Montevideo 11800, Uruguay; E-Mails: veronicacesio@gmail.com (V.C.); heihoracio@gmail.com (H.H.); 3 Department of Pathology, Center for Health Sciences, University of Caxias do Sul, Caxias do Sul, RS 95070-560, Brazil; E-Mail: aeggodoy@gmail.com; 4 Biochemistry Laboratory, Methodist University Center IPA, Porto Alegre, RS 90420-060, Brazil; E-Mail: csfunchal@yahoo.com.br; 5 Department of Microbiology, Parasitology and Immunology, Federal University of Rio Grande do Sul, Porto Alegre, RS 90050-170, Brazil; E-Mail: acoitinho@yahoo.com.br

**Keywords:** yerba mate, polyphenols, antioxidant, nutraceutical

## Abstract

Organic and conventional yerba mate (*Ilex paraguariensis*) is widely used in South America to prepare nonalcoholic drinks rich in polyphenols. These compounds are able to prevent the generation of reactive species, thus minimizing the incidence of several diseases. In this perspective, we hypothesized that yerba mate may have protective effects against pentylenetetrazol (PTZ)-induced oxidative damage in liver and serum of rats. Animals (*n =* 42) received distilled water (control) or yerba mate (organic or conventional) for fifteen days. Then, half of the rats of each group received 60 mg/kg PTZ intraperitoneally or saline solution. After 30 min the animals were euthanized and the liver and blood were collected. The results showed that organic and conventional yerba mate avoided PTZ-induced oxidative damage and nitric oxide production in the liver and serum of the rats. Moreover, both kinds of yerba mate prevented the decrease in enzymatic (superoxide dismutase and catalase) and non-enzymatic (sulfhydryl protein content) defenses in the liver and serum. In addition, histopathologic analysis of the liver showed that yerba mate reduced PTZ-induced cell damage. These findings indicate that yerba mate provides hepatoprotection and improves antioxidant status in the serum, which may contribute to the development of new therapeutic strategies using nutraceuticals drinks.

## 1. Introduction

Yerba mate (*Ilex paraguariensis*, A. Saint Hilaire, Aquifoliaceae) is a widely consumed plant in Southern Latin American countries and has gained rapid popularity in world markets, including in the United States, Asia, and Europe [1,2]. The infusion of the aerial parts of yerba mate is commonly used in different beverages, including *chimarrão* (an infusion of fresh or dried leaves with hot water), *tererê* (an infusion of fresh or dry leaves in cold water) and the mate tea (an infusion of roasted leaves with hot water) [3,4]. In South America, consumption it has been estimated at more than 1 L/day/person of yerba mate drinks [5].

In recent years, research has increasingly emphasized the health properties of mate consumption, such as beneficial effects on glucose metabolism [6]. Yerba mate also presents hypocholesterolemic, vasodilatory, anti-inflammatory [5], anti-obesity [7], and choleretic effects [8], and it is able to reduce the progression of atherosclerosis [9]. Moreover, yerba mate extracts have also been studied in veterinary medicine, presenting anti-*Salmonella* selective activity [10] and angiogenic properties [11]. These effects have been attributed to the bioactive compounds present in yerba mate, such as polyphenols (PPs) and methyl xanthines—mainly caffeine [1,5]. PPs act as hydrogen donators, reducing agents, or singlet oxygen quenchers [12], minimizing the generation of reactive species (RS) implicated in damage of protein, lipids, and nucleic acids [13] thus reducing the incidence of several diseases associated with oxidative stress, including cardiovascular and neurological diseases, cirrhosis, and cancer [14].

Yerba mate can be cultivated according to organic or conventional farming methods. In organic farming, the use of synthetic agrochemicals, hormones, antibiotic agents, and genetic engineering is not allowed whereas in conventional cultivation practice these chemical supplies can be included [15]. Some studies have demonstrated that organic farming can increase synthesis of PPs—secondary metabolites of plants—when compared to conventional farming techniques [16,17].

A previous study from our group [18] demonstrated that both organic and conventional yerba mate were able to reduce oxidative damage in cerebellum, cerebral cortex, and hippocampus of rats treated with pentylenetetrazol (PTZ)—a convulsant drug commonly used in experimental model of epilepsy. However, only the organic yerba mate was able to decrease the PTZ-induced tonic-clonic seizures in these animals. Following this, we aimed to evaluate the effects of organic and conventional yerba mate to prevent oxidative damage on liver and serum of rats treated with PTZ. For this purpose, oxidative damage to lipids and proteins, nitric oxide production, and the enzymatic and non-enzymatic antioxidant defenses (superoxide dismutase, catalase, and sulfhydryl protein content) were evaluated.

## 2. Experimental Section

### 2.1. Chemicals

The reagents PTZ, thiobarbituric acid, 2,4-dinitrophenylhydrazine, 5,5′-dithiobis(2-nitrobenzoic acid), (−)-epinephrine, chlorogenic acid and guanidine hydrochloride were purchased from Sigma-Aldrich (St. Louis, MO, USA). The water used in this study was glass-distilled water (distiller QUIMIS^®^, Quimis Aparelhos Científicos LTDA, Diadema-SP, São Paulo, Brazil). All other reagents (Merck Darmstadt, Alemanha; and Hexapur Amsterdam, Netherlands) and solvents (Nuclear, São Paulo, Brazil) were of analytical grade.

### 2.2. Yerba Mate Samples

Two samples of yerba mate (*Ilex paraguariensis*, St. Hil.) were used: an organically cultivated and properly certified by Instituto Biodinâmico in agreement with international norms; and a conventional sample, both acquired from the local market (Caxias do Sul, Brazil). Samples were composed of ground dried leaves (without stems or branches). The mate drinks were prepared as *chimarrão*, using 20 g of yerba/100 mL of distilled water at 90 °C for 90 s, simulating the traditional use by the general population. After cooling to 25 °C, the samples were filtered using Millipore equipment (pore size, 0.45 µm; catalog number SFGS 047LS, Millipore Corp., São Paulo, Brazil). The obtained mate drinks were freeze-dried (Liobras, model L101 freeze dryer, São Paulo, Brazil) at 40 °C, 10^−1^ bar, stored at −20 °C and solubilized in distilled water immediately before use. Total phenolic content of both mate drinks was determined by the Folin-Ciocalteau assay modified by Singleton *et al.* [19]. The polyphenolic content of organic yerba mate was 322.0 ± 4.24 mg of chlorogenic acid equivalents/100 g of dry weight, and 319.0 ± 7.07 mg of chlorogenic acid equiv./100 g of dry weight for the conventional one. The main PPs found in both yerba mates were chlorogenic acid, acyl derivatives of phenolic acids and the flavonoid rutin [18].

### 2.3. Animals and Treatment

In order to avoid a hormonal influence on the biological response, only male Wistar rats were used (*n =* 42, with 3 months old, weighing 250–300 g) from a breeding colony of Centro Universitário Metodista (Porto Alegre, Brazil). They were maintained at a temperature of 22–24 °C, on a 12 h light/12 h dark cycle, with free access to food and water. The experiments were performed in accordance with the “Guide for the Care and Use of Laboratory Animals, DHEW, publication no. (NIH) 85-23, 1985” and approved by the local ethical committee IPA 439/2009. Animals were randomly allocated to one of the three experimental groups (*n =* 14 per group): group 1 served as control, and received distilled water; groups 2 and 3 received, by gavage, the organic and conventional mate drinks (50 mg/kg of body weight) respectively once a day for 15 days. On the 15th day, half of the rats of each group (*n =* 7) received intraperitoneally (i.p.) a single dose of PTZ (60 mg/kg of body weight) dissolved in sterile isotonic saline. The other half of the rats of each group (negative control, group 1) received only saline solution (i.p.). After 30 min, animals were euthanized by decapitation, and the blood and liver were collected. Serum samples were obtained from the blood by centrifugation (5 min at 3000× *g*). The liver was washed three times with iced 1.5% KCl to remove all blood. The liver and serum were stored at −80 °C until biochemical analysis. Slices from the liver were used for histopathological analysis. Before each biochemical assay, liver samples were homogenized in phosphate buffered saline (pH 7.4) using a ground-glass-type Potter-Elvehjem homogenizer, and centrifuged for five min. Supernatant was used in the assays and all processes were performed under cold conditions.

### 2.4. Protective Effects of Yerba Mate in Liver and Serum of Rats

Oxidative damage to lipids and proteins, nitric oxide (NO^•^) production and the antioxidant enzyme activities (superoxide dismutase and catalase) were analyzed. The protein sulfhydryl content was assessed as a non-enzymatic cellular defense. Oxidative damage to lipids was determined by a method that was based on the reaction with thiobarbituric acid reactive species (TBARS) during an acid-heating reaction. This sensitive method is widely adopted for measuring lipid peroxidation, as previously described [20]. The results were expressed as nmol/mg of protein. Oxidative damage to proteins was measured by determination of the carbonyl group by a reaction with dinitrophenylhydrazine (DNPH), according to Levine *et al.* [21]. DNPH reacts with protein carbonyls to form hydrazones that can be measured spectrophotometrically. The results were expressed as nmol DNPH/mg of protein. NO^•^ measurements are very difficult to access in biological specimens. Therefore, the Griess reaction was used to determine the tissue’ nitrite (NO^−2^) as an index of NO^•^ production [22], according to Green [23]. The results were expressed as nmol/mg protein.

Superoxide dismutase (EC 1.15.1.1) (SOD) activity was determined according to Bannister and Calabrese [24] and results were expressed as USOD/mg of protein. One unit of SOD is defined as the amount of enzyme that inhibits the rate of adrenochrome formation by 50%. Catalase (EC 1.11.1.6) (CAT) activity was determined by hydrogen peroxide (H_2_O_2_) decomposition rate, according to Aebi [25]. The values were expressed as mmol of H_2_O_2_/minute/mg of protein. Protein sulfhydryl content was determined by a reaction with 5,5′-dithiobis(2-nitrobenzoic acid) (DTNB), according to Askenov and Markesbery [26] and results were expressed as nmol DTNB/mg of protein. Protein concentration was measured using Bradford method [27] for the liver tissues, and the Total Proteins kit from Labtest (Protein Kit, Labtest Diagnostica S.A., Brazil) for the serum. All assays were performed in triplicate.

### 2.5. Histopathological Analysis of the Liver Tissues

Seven micrometer thick paraffin sections of buffered formalin-fixed liver samples were stained with hematoxylin-eosin (H & E), according to Gamble [28], for photomicroscopic observation of liver histological architecture from control group and treated rats.

### 2.6. Statistical Analysis

The number of animals used in this study was determined by the statistical F-test multivariate analysis of variance (MANOVA) (*F* = 3.21, α = 0.05, power = 90%). Values are presented as mean ± standard error of the mean (S.E.M.). Results were subjected to one-way ANOVA followed by Tukey’s post-hoc test (*p* ≤ 0.05). Data were analyzed using SPSS 18.0 software (SPSS Inc., Chicago, IL, USA).

## 3. Results and Discussion

In the present work, the effects of organic and conventional yerba mate were evaluated on liver and serum of Wistar rats subjected to PTZ-induced oxidative damage. As expected, PTZ treatment induced an increase in lipid peroxidation and oxidative damage to proteins, and increased in nitric oxide production both in liver (Table 1) and serum (Table 2) of the rats. Additionally, activities of SOD and CAT, and sulfhydryl protein content were significantly decreased in PTZ group, which indicated depletion of the antioxidant system in the liver (Table 1), and serum (Table 2).

**Table 1:** Oxidative stress markers in the liver of rats treated with yerba mate.

Group	TBARS (nmol/mg of protein)	Carbonyl Protein (nmol DNPH/mg of protein)	Nitric Oxide (nmol/mg of protein)	Superoxide Dismutase (U SOD/mg of protein)	Catalase (mmol H_2_O_2_/min/mg of protein)	Sulfhydryl Protein (nmol DTNB/mg of protein)
Control	1.30 ± 0.11 #	1.62 ± 0.16 #	1.14 ± 0.22 #	13.37 ± 1.12 #	70.10 ± 1.20 #	0.35 ± 0.01 #
Pentylenetetrazol (PTZ)	3.53 ± 0.02 *	4.77 ± 0.15 *	3.43 ± 0.17 *	1.58 ± 0.11 *	43.24 ± 0.93 *	0.11 ± 0.02 *
Organic yerba mate	1.33 ± 0.09 #	1.49 ± 0.12 #	1.09 ± 0.06 #	15.28 ± 0.20 #	73.82 ± 1.17 #	0.49 ± 0.01 #*
Conventional yerba mate	1.24 ± 0.05 #	1.55 ± 0.09 #	0.94 ± 0.18 #	16.17 ± 1.14 #	77.70 ± 1.09 #	0.47 ± 0.03 #*
Organic yerba mate + PTZ	1.41 ± 0.11 #	1.73 ± 0.18 #	1.20 ± 0.08 #	10.73 ± 0.18 #	60.39 ± 2.14 #*	0.40 ± 0.02 #
Conventional yerba mate + PTZ	1.36 ± 0.33 #	2.12 ± 0.19 #	1.12 ± 0.07 #	8.19 ± 0.75 #*	66.00 ± 1.13 #*	0.29 ± 0.03 #

Data are mean ± standard error of the mean S.E.M. values. * Values significantly different from the control group; # Values significantly different from PTZ group. ANOVA and Tukey’s post-hoc test (*p* ≤ 0.05) were used. DNPH: Dinitrophenylhydrazine; SOD: Superoxide dismutase; DTNB: 5,5′-dithiobis(2-nitrobenzoic acid).

**Table 2:** Oxidative stress markers in the serum of rats treated with yerba mate.

Group	TBARS (nmol/mg of protein)	Carbonyl Protein (nmol DNPH/mg of protein)	Nitric Oxide (nmol/mg of protein)	Superoxide Dismutase (U SOD/mg of protein)	Catalase (mmol H_2_O_2_/min/mg of protein)	Sulfhydryl Protein (nmol DTNB/mg of protein)
Control	1.74 ± 0.08 #	1.59 ± 0.24 #	1.89 ± 0.22 #	5.30 ± 0.70 #	13.52 ± 0.57 #	0.43 ± 0.07 #
Pentylenetetrazol (PTZ)	2.44 ± 0.06 *	3.78 ± 0.10 *	5.13 ± 0.06 *	0.25 ± 0.02 *	7.20 ± 0.70 *	0.07 ± 0.01 *
Organic yerba mate	1.79 ± 0.13 #	1.60 ± 0.15 #	2.12 ± 0.40 #	8.06 ± 0.31 #	15.06 ± 1.02 #	0.50 ± 0.10 #
Conventional yerba mate	1.76 ± 0.04 #	1.48 ± 0.17 #	2.02 ± 0.56 #	7.19 ± 0.36 #	14.53 ± 0.70 #	0.46 ± 0.03 #
Organic yerba mate + PTZ	2.03 ± 0.19 #	1.93 ± 0.21 #	2.59 ± 0.23 #	6.98 ± 0.54 #	13.58 ± 1.31 #	0.43 ± 0.02 #
Conventional yerba mate *+*PTZ	1.90 ± 0.17 #	2.05 ± 0.18 #	2.26 ± 0.72 #	5.13 ± 0.77 #	11.10 ± 0.90 #	0.34 ± 0.06 #

Data are mean ± S.E.M. values. * Values significantly different from the control group; # Values significantly different from PTZ group. ANOVA and Tukey’s post-hoc test (*p* ≤ 0.05) were used.

Organic and conventional yerba mate alone did not induce oxidative damage to lipids and proteins or increase nitric oxide production in liver and serum of rats. In the liver, both organic and conventional yerba mate were able to increase sulfhydryl protein content (Table 1). Pre-treatments with organic and conventional yerba mate prevented against oxidative damage to lipids and proteins, and avoided the increase in nitric oxide production induced by PTZ in the liver (Table 1), and serum of rats (Table 2). In addition, the treatments with both types of yerba mate minimized the decrease in SOD and CAT activities, and in sulfhydryl protein content induced by PTZ, both in liver (Table 1) and serum (Table 2).

**Figure 1:**
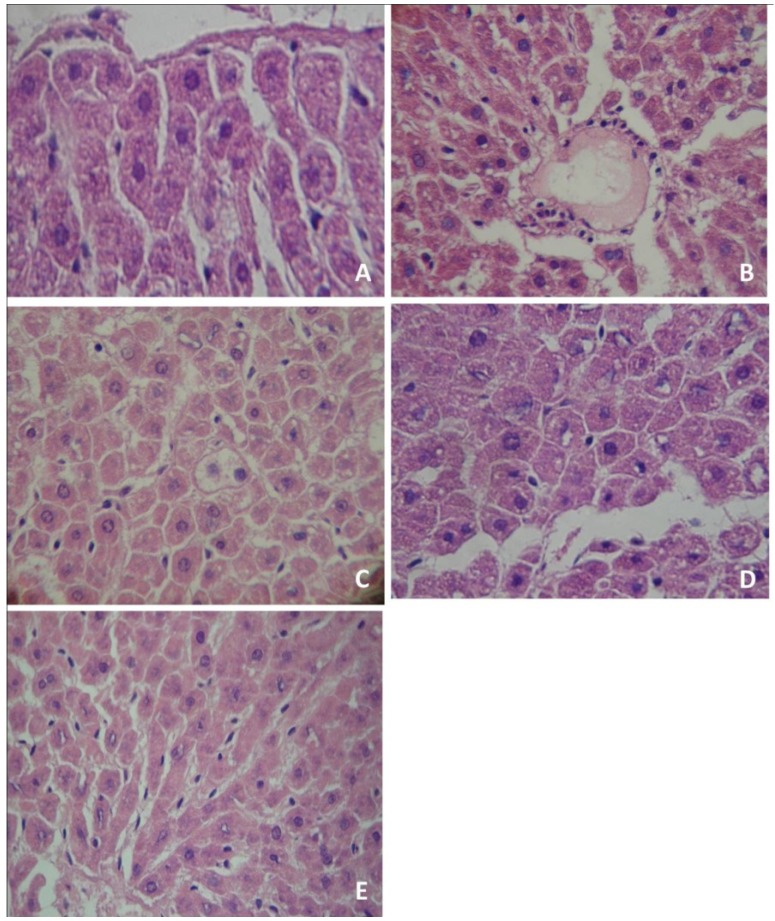
Histological analysis of rat liver tissues. Tissues were stained with hematoxylin-eosin and analyzed using a light microscope and a 400× oil immersion objective. (**A**) control group; (**B**) Pentylenetetrazol administration produced infiltration of inflammatory cells (IIC); (**C**) Pentylenetetrazol administration produced hepatic steatosis (HS); (**D**) organic yerba mate treatment (50 mg/kg) plus pentylenetetrazol prevented infiltration of inflammatory cells (IIC) and hepatic steatosis (HS); (**E**) conventional yerba mate treatment (50 mg/kg) plus pentylenetetrazol prevented IIC and HS. (**A**, **D** and **E**: normal hepatocytes). The photomicrographs show the most representative slide of each group.

The morphological evaluation of liver slices showed that the control group had normal characteristics, with expected glycogen amount and no evidence of suffering (Figure 1A). Similar findings were observed in rats treated with organic and conventional yerba mate, which presented normal cellular architectures in the liver (data not shown). Conversely, PTZ administration induced liver damage, with infiltration of inflammatory cells (Figure 1B) and evidence of steatosis (Figure 1C). The pre-treatment with organic (Figure 1D) or conventional (Figure 1E) yerba mate markedly reduced liver damage caused by PTZ, with no evidence of suffering.

*I. paraguariensis* is an important source of PPs, presenting higher levels of these compounds than those related for red wines and green tea, which is touted as having a very high antioxidant capacity [29]. In this study, we investigated the ability of yerba mate, as usually consumed, to reduce oxidative damage to liver and serum of rats treated with PTZ, a drug able to induce an intense oxidative stress through the generation of superoxide anion and hydroxyl radicals [22].

The administration of both organic and conventional yerba mate drinks to rats for fifteen days avoided the increase in oxidative damage to lipids, proteins and the nitric oxide production induced by PTZ, both in liver and serum of rats. Damage to lipids and proteins, as well as high amounts of nitric oxide, affect the cellular redox balance. Increased concentrations of nitric oxide can easily react with superoxide anion generating peroxynitrite, which can rapidly cause protein nitration or nitrosylation, and lipid peroxidation, leading to cell death [30]. In addition, peroxynitrite is a potent tissue-damaging, which has a high affinity for sulfhydryl groups, and thus inactivates several key sulfhydryl-bearing enzymes [31].

It has already been shown that the administration of 1–2 g/kg of instant mate for 60 days can reduce oxidative damage to lipids in liver and serum of Swiss mice [8]. In our study, a similar result was found using low doses and short treatment time of *I. paraguariensis*, which are important data for a nutraceutical approach.

Organic and conventional yerba mate administration also prevented the decrease of enzymatic and non-enzymatic antioxidant defenses in liver and serum of rats. The enzyme superoxide dismutase catalyzes the dismutation of superoxide anions producing H_2_O_2_, which can be eliminated by the action of the catalase. It was previously shown that *I. paraguariensis* is a potent scavenger of the superoxide anion [32], corroborating the results found in our work. Thiols groups (represented, mainly by sulfhydryl proteins) protect molecules from excessive production of RS by hydrogen donation, restoring damaged molecules [33]. Together, enzymatic and non-enzymatic antioxidant defenses can act to minimize the risk of various systemic diseases associated with oxidative stress, such as atherosclerosis, neurodegenerative disorders, and cancer [14,34].

The protective effect of yerba mate on the liver was corroborated by histopathological analysis, which demonstrated no cellular damage in rats treated with yerba mate drinks plus PTZ. It was previously shown that yerba mate could help to protect unsaturated fatty acids from oxidation, and thus, prevent against hepatocyte fragility in Swiss [8] and Wistar rats [32]. Moreover, it has been demonstrated that mate tea was able to avoid hepatic fatty acids deposition and steatosis in Sprague-Dawley rats [35]. Chlorogenic acid, the main polyphenol in yerba mate, is thought to reduce the risk of cardiovascular disease by decreasing the oxidation of low-density lipoprotein (LDL) and cholesterol [36], allowing it to have a protective effect against steatosis. In addition, the alkaloid caffeine, another bioactive component of yerba mate, is also effective in suppressing fat accumulation in the liver, thus improving hepatic lipid metabolism [37]. Liver injury is associated with alteration of important metabolic functions, impairing the body’s homeostasis. Nutraceutical compounds that are able to prevent or reduce oxidative damage are potential candidates to new studies in antioxidant research field.

Biological effects of yerba mate are well known, and some studies have demonstrated its potential benefits to the cardiovascular system [32] and against DNA double-strand breaks [6,38]. Our results are consistent with these previous researches and show that yerba mate can improve the redox metabolic status of the body. Additionally, although it was previous related different biological effect for organic and conventional yerba mate [18], in our present study both samples presented similar results. Furthermore, it is important to mention that no difference was observed in the total phenol content of organic and conventional yerba mate studied in our work which can explain, at least in part, the results obtained by us.

## 4. Conclusions

Our results demonstrate that both organic and conventional yerba mate present significant hepatic and systemic protection against PTZ-induced oxidative damage in rats. Therefore, further studies are needed to provide a better understanding about the mechanisms of action of yerba mate in oxidative stress. Additionally, it would be interesting to study the isolate compounds founded in *I. paraguariensis*, which could help to elucidate the main bioactive molecules.
